# Combating Prozone Effects and Predicting the Dynamic Range of Naked-Eye Nanoplasmonic Biosensors through Capture Bioentity Optimization

**DOI:** 10.1021/acsmeasuresciau.4c00010

**Published:** 2024-05-08

**Authors:** Zoe Bradley, Nikhil Bhalla

**Affiliations:** † Nanotechnology and Integrated Bioengineering Centre (NIBEC) School of Engineering, Ulster University, 2-24 York Street, Belfast BT15 1AP, Northern Ireland; ‡ School of Engineering, Ulster University, Healthcare Technology Hub, 2-24 York Street, Belfast BT15 1AP, U.K.

**Keywords:** biosensors, hook-effect, nanoplasmonics, mathematical-modeling, lateral flow devices, gold nanoparticles

## Abstract

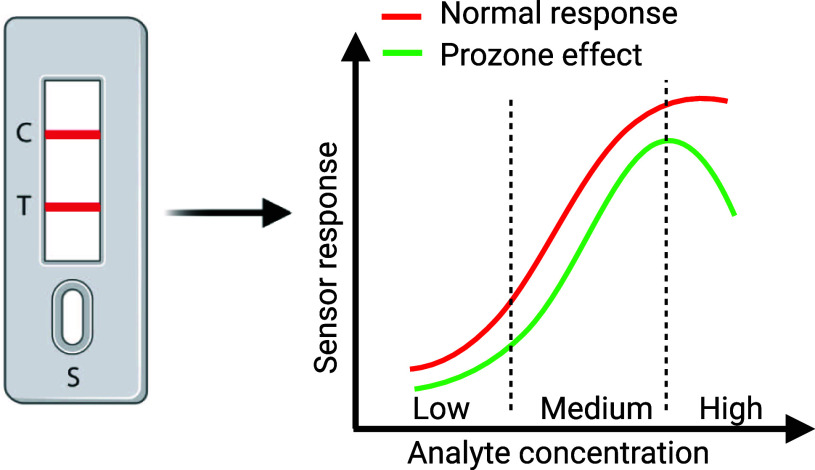

Accurately quantifying high analyte concentrations poses a challenge due to the common occurrence of the prozone or hook effect within sandwich assays utilized in plasmonic nanoparticle-based lateral flow devices (LFDs). As a result, LFDs are often underestimated compared to other biosensors with concerns surrounding their specificity and sensitivity toward the target analyte. To address this limitation, here we develop an analytical model capable of predicting the prozone effect and subsequently the dynamic range of the biosensor based on the concentration of the capture antibody. To support our model, we conduct a sandwich immunoassay to detect C-reactive protein (CRP) in a phosphate-buffered saline (PBS) buffer using an LFD. Within the experiment, we investigate the relationship between the CRP dynamic range and the prozone effect as a function of the capture antibody concentration, which is increased from 0.1 to 2 mg/mL. The experimental results, while supporting the developed analytical model, show that increasing the capture antibody concentration increases the dynamic range. The developed model therefore holds the potential to expand the measurable range and reduce costs associated with quantifying biomarkers in diverse diagnostic assays. This will ultimately allow LFDs to have better clinical significance before the prozone effect becomes dominant.

## Introduction

The hook effect, also known as the prozone effect, is a phenomenon that commonly occurs in antibody-based sandwich immunoassay biosensors.^1,2^ In a typical immunoassay, the binding of antibodies to the analyte leads to the formation of a visible signal, such as a color change or a fluorescent signal where the intensity of this signal is directly proportional to the concentration of the analyte in the sample being tested.^3,4^ However, the prozone effect happens when the concentration of the analyte becomes so high that it exceeds the capacity of the antibodies in the assay.^5^ In this situation, the excess analyte can saturate or overwhelm the binding sites on the antibodies, and as a result, the sensor response is inhibited, leading to a false-low or even false-negative test result.^6^

Plasmonic nanoparticle-based lateral flow devices (LFDs) are simple, low-cost, and portable diagnostic tests used at the point of care (POC).^7^ LFDs typically exhibit a qualitative or semiquantitative dose response, meaning the intensity of the test signal can provide an indication of the concentration of the target analyte in the sample.^8^ So far, LFDs are trusted more as a qualitative device than a device that reliably provides quantitative measurement of the biomarker under investigation. This is primarily due to the issues surrounding accuracy and sensitivity as they often incorporate sandwich immunoassays, which is associated with the prozone effect, such as for the detection of human chorionic gonadotropin (hCG) used for pregnancy tests and beta-trace protein to detect cerebrospinal fluid (CSF) leakage during spine surgery.^9,10^ The presence of the prozone effect reduces the immunoassay dynamic range, which can therefore limit the ability to detect the analyte within its clinical range.^11^

To overcome the prozone effect, strategies such as diluting the sample prior to use^12^ or computational modeling of test and control lines using real-time kinetics^13^ have been used to accurately measure high analyte concentrations. Previous work by Ross et al. used Python to record the real-time kinetics of a LFD assay and used the brightness of the test line over control line ratio to determine the analyte concentration. Presence of the prozone effect was observed when the LFD displayed a reduced signal development on the control line with a rapid signal development on the test line or when there was a decrease in signal development on either line for the first 10 min, followed by an increase in the intensity of signal development on the control line for the remainder of the assay duration.^14^ Other research by Shin et al. monitored how changing the concentration of the capture antibody can help to overcome the prozone effect by expanding the dynamic range. A regression analysis was used to compare the known analyte concentration vs wavelength response alongside other statistical modeling to investigate linearity, prozone effect, and precision.^15^

In particular, many previous works have demonstrated the presence of the prozone effect in C-reactive protein (CRP) as a case study. CRP is a well-investigated inflammatory biomarker, that is, an acute-phase protein produced by the liver, and rises rapidly in the instance of inflammation. CRP can help to diagnose various illnesses such as sepsis, cardiovascular disease, and arthritis.^16,17^ For instance, in the case of sepsis, CRP levels <10 μg/mL are considered normal with elevated levels reaching 40–200 μg/mL.^18^ However, many previous sepsis studies deflect from the prozone effect presence by detecting CRP in a ng/mL dynamic range, which is not practical in a clinical setting.^19^

**Figure 1:**
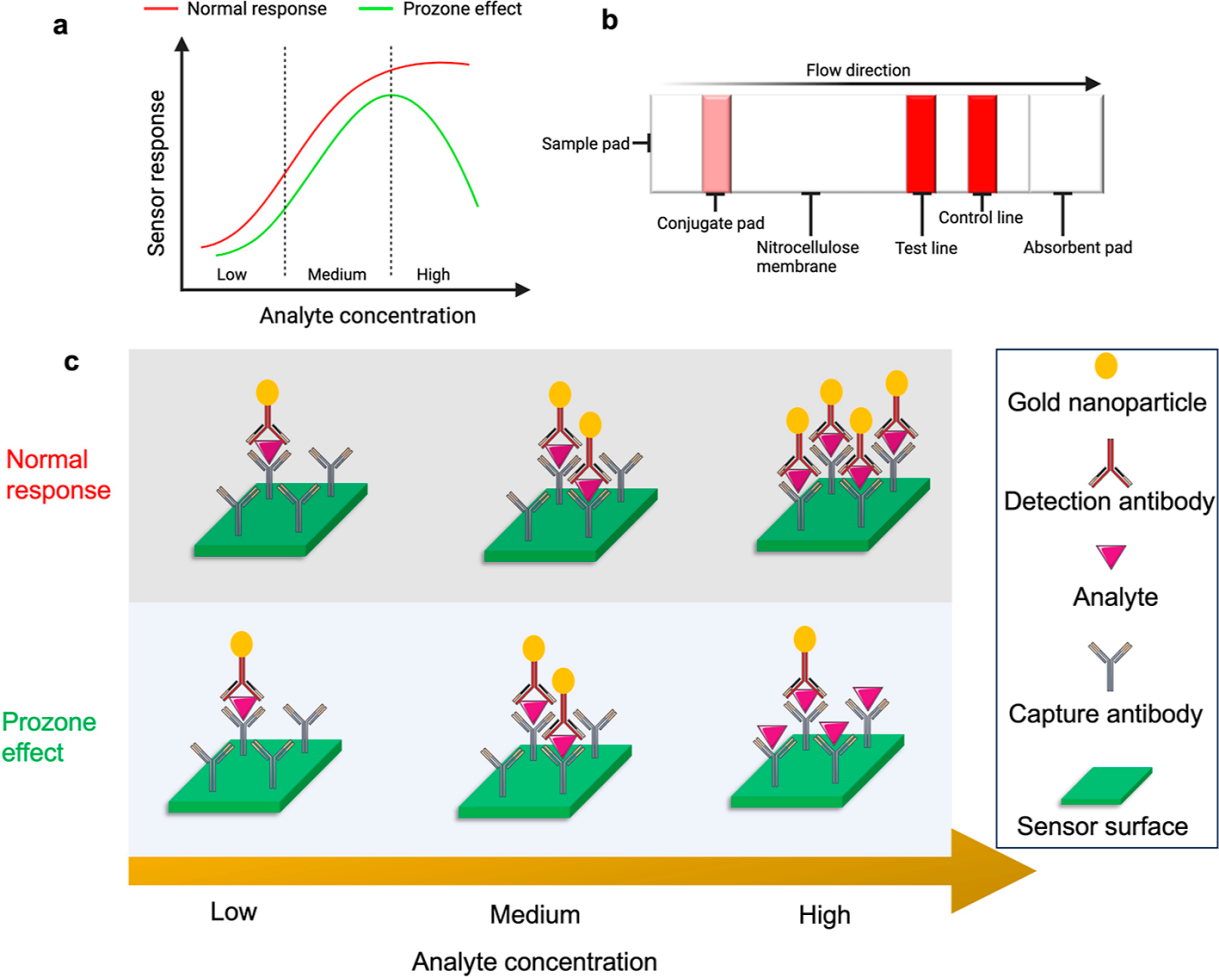
Prozone effect: (a) typical normal immunoassay response compared to a response with the prozone effect present; (b) components of a lateral flow device (LFD), which utilizes naked-eye nanoplasmonic assays and often suffers from the prozone effect; and (c) sensor surface state at the test line when exposed to low, medium, and high analyte concentrations under normal and prozone-affected conditions.

The prozone effect, often associated with the saturation of the detection antibody, can also be influenced by limitations in the capture antibody. In LFDs where the interaction between the analyte and detection antibody happens in solution with an extended contact time, in this case 20 min, the impact of the capture antibody becomes more pronounced. This is because the interaction with the capture antibody occurs rapidly in heterogeneous phases, which can lead to reduced sensitivity or anomalous results if the amount of the capture antibody is insufficient. Our work investigates how the change in capture antibody concentration can help overcome the prozone effect in immunoassays; see the schematics in Figure 1 that displays a sensor response seen by the prozone effect compared to a normal immunoassay sensor response.

As a standard test system, we have used CRP and its antibody (0.1, 0.5, 1, 1.5, and 2 mg/mL) in our experiments. To explain our observations, we developed a new model to calculate the range of detection. By optimizing the concentration of the capture antibody, the assay’s dynamic range can be improved and the impact of the prozone effect is minimized. To calculate the dynamic range, we develop an asymmetric equation that calculates the inflection point, *C*, that is, the analyte concentration at a 50% sensor response rate. The inflection point is then used to determine *C*_90_ (analyte concentration at a 90% sensor response rate) and *C*_10_ (analyte concentration at a 10% sensor response rate) to evaluate the dynamic range for each capture antibody concentration to better understand the prozone effect kinetics upon changing the capture antibody concentration.

## Results and Discussion

**Figure 2:**
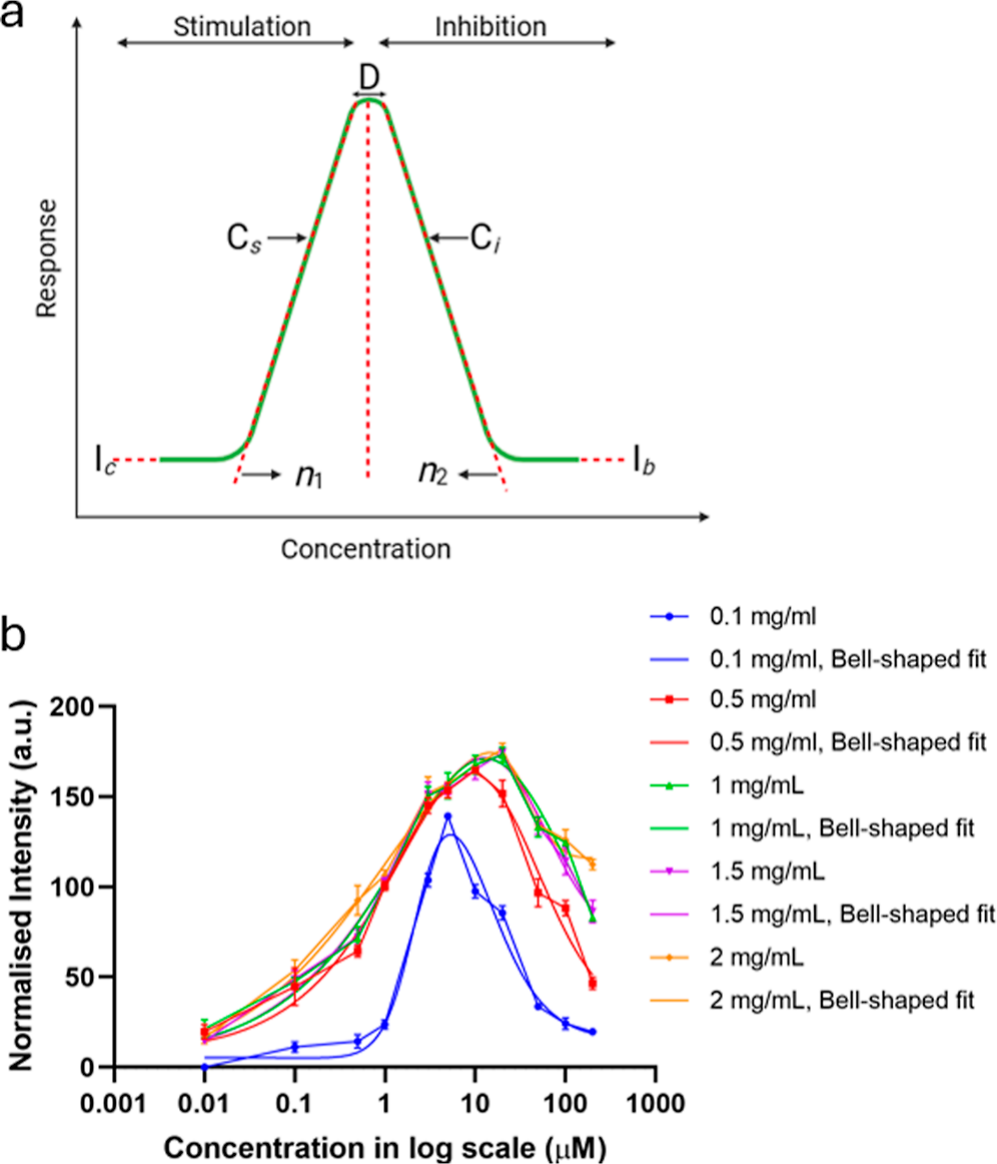
Sensor response with the prozone effect: (a) concentration vs bell-shaped curve response displaying *I*_c_ and *I*_b_, which represent the intensity at the two ends of the curve. *C*_s_ and *C*_i_ are half-maximal stimulatory and inhibitory concentrations, *D* is the plateau in the middle of the curve, and *n*1 and *n*2 are hill slope factors. (b) CRP concentrations 0.01–200 μg/mL displaying the presence of the prozone effect, fitted with a bell-shaped fit.

The concept of concentration–response curves, often depicted as bell-shaped curves, is fundamental in sensing, biology, and chemistry to understand how the concentration of a substance (e.g., a drug or a signaling molecule) relates to its biological effect. These curves help researchers quantify the response of a system to varying concentrations of a compound, as seen in Figure 2a.

Figure 2b shows the sensor response (intensity of colorimetric signal) upon change in the CRP concentration at capture antibody concentrations of 0.1, 0.5, 1, 1.5, and 2 mg/mL. From Figure 2b, we also observe a dual response-stimulation and inhibition activity exhibited by the prozone effect present in the immunoassay. Essentially, the prozone effect appears at 5 μg/mL for a 0.1 mg/mL capture antibody concentration but does not start until 20 μg/mL for 1 and 2 mg/mL capture antibody concentrations.

To further understand the sensor response, we fit the response in Figure 2b with a bell-shaped curve using eq 1.
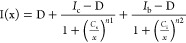
1Here, *I*_c_ and *I*_b_ are the plateaus at the left and right ends of the curve, i.e., intensity at the two ends of the curve. *D* is the plateau level, intensity, in the middle of the curve. *C*_S_ and *C*_i_ are half-maximal stimulatory and inhibitory concentrations. From a mathematical perspective, *C*_S_ and *C*_i_ are inflection points of the stimulatory and inhibitory parts of the bell-shaped curve. *n*1 and *n*2 are unitless slope factors or more generally known as hill slopes.

**Figure 3:**
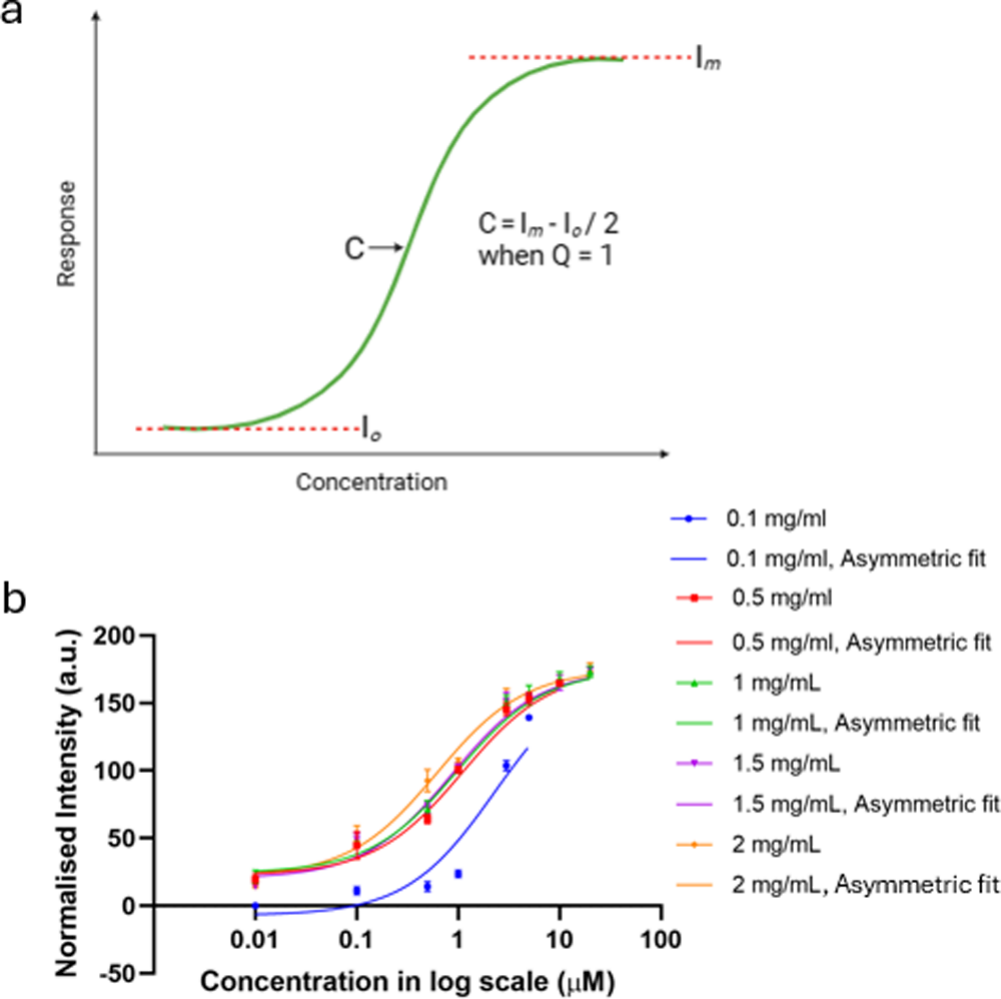
Preprozone effect sensor response: (a) concentration vs the stimulatory response showing *I*_o_ and *I*_m_ as the initial and maximum values of the intensity; *Q* is an asymmetry factor and *C* provides the value of concentration at which the sensor achieves its 50% response. (b) CRP concentrations before the prozone effect becomes present in the dose response of the immunoassay, fitted with an asymmetric curve.

To extract more features of the response, up to those concentrations where the saturation starts (just before the prozone effect starts), see Figure 3a (for model) and b, where the data are fitted with an equation (asymmetrical) that incorporates a new factor, *C*. This factor *C* is the inflection point, which is defined as the point where the curvature of the fitted line changes direction or sign.
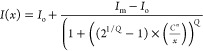
2Here, *I*_o_ and *I*_m_ are the initial and maximum values of the intensity, and *Q* is an asymmetry factor. In the context of an immunoassay dose–response, as in here, *C* can provide the value of concentration at which the sensor achieves its 50% response, i.e., (*I*_m_ – *I*_o_)/2 when *Q* = 1. This is because when *Q* = 1, there is a symmetrical curve around the inflection point. *n* is the steepness factor or slope of the curve, which is considered as 1 in our case. If the response of the sensor was decreasing, then this slope could be considered at −1.

The variations in the inflection point, *C* and standard error of the mean (SEM) in the data in Figure 3b, have been experimentally determined by multiple replicates of the experiments. SEM was calculated to better understand the reliability of the sample mean. The *C* values, *C*_10_ and *C*_90_, with corresponding error values can be determined from eqs 3 and 4, as seen in Table 1. Since *C* is the concentration that leads to 50% sensor response, we can calculate concentrations where response of the sensor varies from 0 to 100%, *p*-values, using the following eq 3
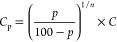
3

**Table 1:** Inflection Point *C*, *C*_10_ and *C*_90_, with ±SEM Values for Each CRP Capture Antibody Concentration (0.1, 0.5, 1, 1.5, and 2 mg/mL)

CRP concentration (mg/mL)	0.1	0.5	1	1.5	2
*C*	2.286 (1.19, 3.38)	1.105 (0.95, 1.26)	0.981 (0.87, 1.09)	0.873 (0.78, 0.97)	0.620 (0.56, 0.68)
*C*_10_	0.254 (0.13, 0.38)	0.123 (0.11, 0.14)	0.109 (0.10, 0.12)	0.097 (0.09, 0.11)	0.068 (0.06, 0.08)
*C*_90_	20.574 (10.70, 30.40)	9.945 (8.57, 11.32)	8.828 (7.85, 9.80)	7.857 (6.99, 8.73)	5.580 (5.02, 6.13)

Again, since the value of *n* is 1, eq 3 can be rewritten as eq 4 below
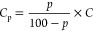
4

Table 1 indicates that our assay is more reliable at lower analyte concentrations. This is because the large *C*_10_ and *C*_90_ values, shown by the lower CRP capture antibody concentrations, indicate that a higher concentration of a substance is required to produce a specific response in the assay. Moreover, the inflection point, *C*, represents the half-maximal effective concentration of the response. A lower value for *C* indicates greater potency, meaning that a lower concentration of the substance is required to produce a desired response, which is seen as the CRP capture antibody concentration increases. *C*_10_ and *C*_90_ describe the concentration of a substance that produces a response that is 10 and 90% of the maximum or desired effect, respectively.^20^ From a qualitative perspective, from the data shared in Figure 3b and Table 1, we can observe that increasing the capture antibody concentration can help overcome the prozone effect by improving the detection range of the analyte. The capture antibody is responsible for binding the analyte, while the detection antibody is labeled with a signal-generating molecule, in our case gold nanoparticles (AuNPs), that allows for the measurement or detection of the analyte.^21,22^ In the presence of very high analyte concentrations, the available capture antibodies may become saturated with the analyte, limiting their ability to bind to additional analyte molecules.

**Figure 4:**
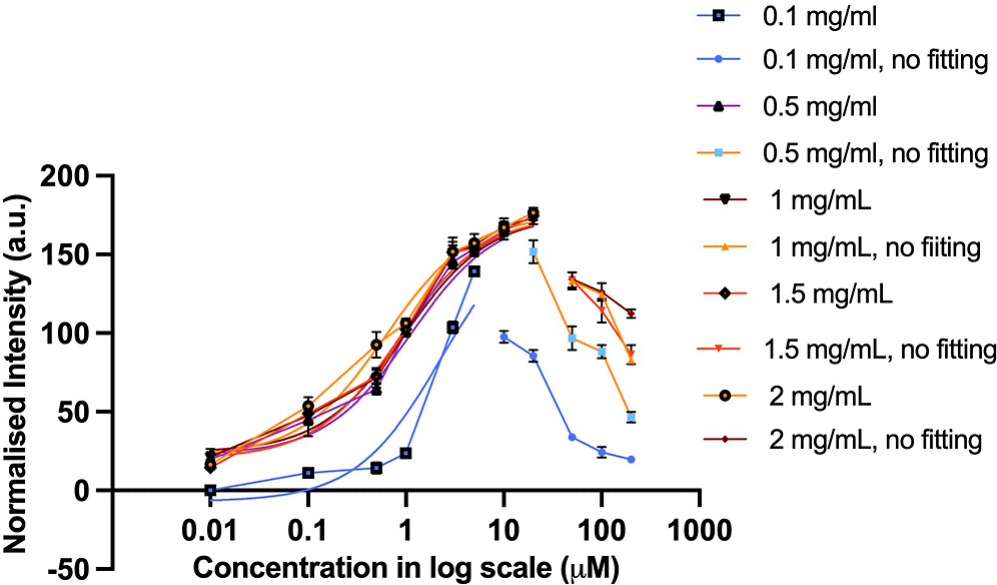
Prozone effect analysis: combined graph of data used for the asymmetric curve in Figure 3b and the data that were not fitted with the asymmetric curve (used from Figure 2b) representing the sensor signal measured from different CRP detection, using antibody concentrations ranging from 0.01 to 200 μg/mL.

Figure 4 is a combination of Figure 3b fitted with eq 2 and prozone effect data not used from Figure 2b as this inhibition activity cannot be used to accurately determine the assay dynamic range. The dynamic range is a term used to describe the range of concentrations over which an assay can provide accurate results. A large dynamic range is most desirable as it allows for the detection and measurement of both low and high concentrations of the analyte accurately. A larger dynamic range also provides more flexibility in experimental design and allows for the detection of subtle changes in response at low concentrations as well as the assessment of saturation or plateau effects at high concentrations.^23^

Enhancements in the dynamic range of biosensors have profound implications for reagent consumption and associated costs. A broader dynamic range enables biosensors to accurately detect a wider span of analyte concentrations without saturation or a loss of sensitivity. This improvement allows for the use of smaller reagent volumes per assay as smaller sample and reagent quantities suffice to cover the desired concentration range. Moreover, optimized reagent concentrations can be achieved, reducing the consumption of costly reagents while maintaining accuracy. Biosensors with extended dynamic ranges streamline assay procedures, minimizing the need for multiple dilutions or repeated assays, thereby reducing reagent waste. The increased efficiency and throughput afforded by these advancements lead to lower overall costs despite potential initial investments in the technology. Consequently, the broader accessibility and cost-effectiveness of biosensor technology are augmented, benefiting diverse applications ranging from medical diagnostics to environmental monitoring.

To compute the dynamic range of the sensor, we consider two points *C*_10_ and *C*_90_, i.e., the concentrations that lead to 10 and 90% maximal response. The difference between these values provides the working range of the sensor; see eq 5.
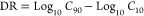
5

In an anomalous situation when there is an abrupt change in the sensor signal, i.e., a drop in the sensor signal without reaching the saturation (plateau) predicted by parameter *D* in eq 2, the maximum intensity value observed within the experiments should be considered as *C*_90_. In our experiments, this exceptional sensor response is observed for 0.1 mg/mL antibody concentration, for which *C*_90_ is considered as 5 μM instead of the minimum value of 9.97 μM calculated by eq 4. Such an exceptional response can also easily be indicated by observing standard deviation in the mean of the *C* calculated from eq 3 for different values of experimental replicates. Essentially, if the mean has a large standard deviation, the peak experimental value can be considered as *C*_90_ as this value is within the linear range of the sensor response.

**Figure 5:**
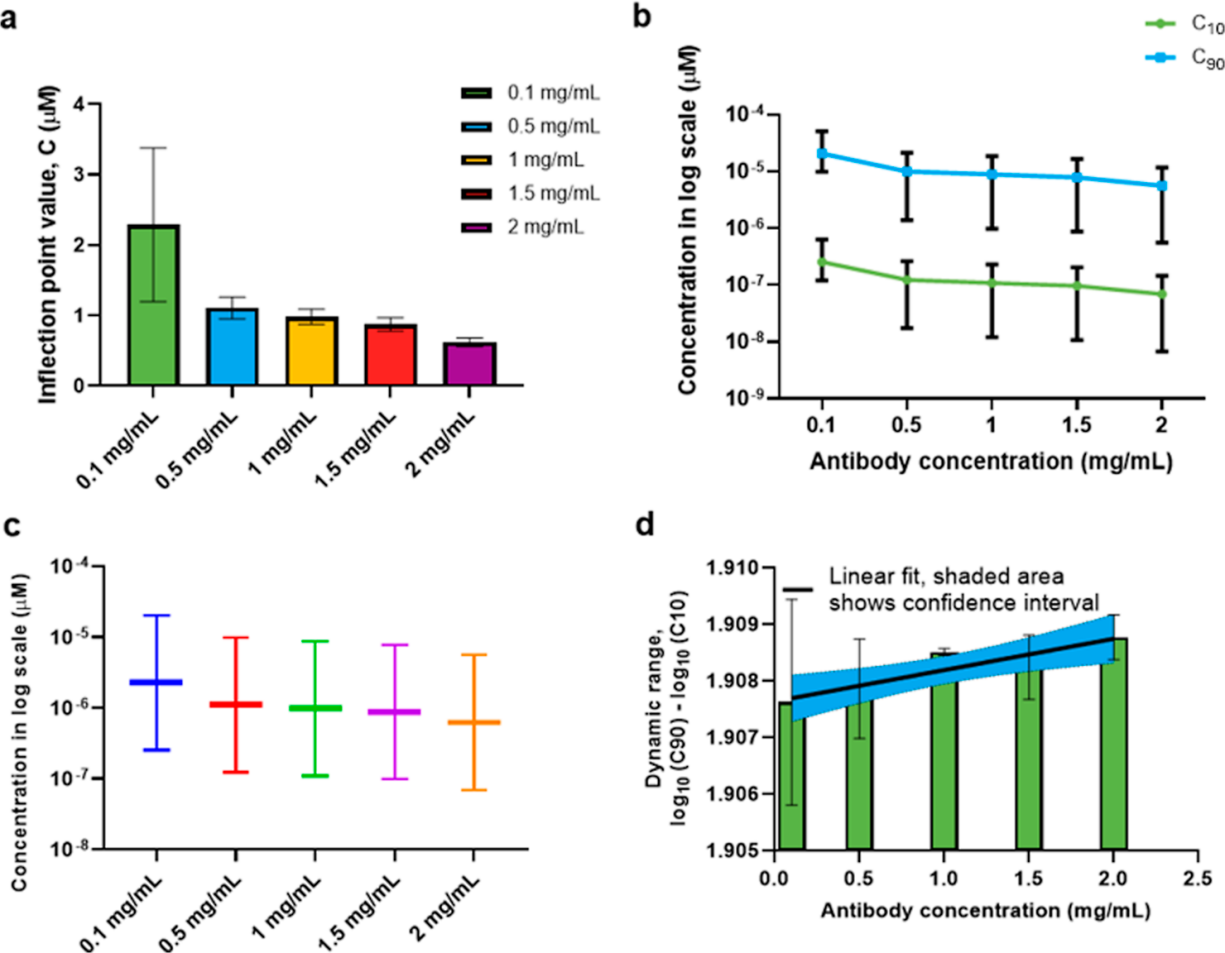
Statistical analysis: (a) inflection points plotted with SEM bars for each capture CRP antibody concentration; (b) *C*_10_ and *C*_90_ plotted for each CRP capture antibody concentration; (c) *C*_10_ and *C*_90_ values plotted for each CRP capture antibody concentration, and (d) dynamic range plotted for each CRP capture antibody concentration.

Figure 5a used the data in Table 1 to plot the inflection point *C* for each capture antibody concentration. As the capture antibody concentration increases, the inflection point value decreases and SEM values improves. Figure 5b shows that as the CRP capture antibody concentration increases, the *C*_10_ and *C*_90_ values decrease. *C*_10_ and *C*_90_ values with SEM values have been plotted in Figure 5c from which the assay dynamic range has been plotted for each CRP capture antibody concentration, as seen in Figure 5d. From Figure 5d, we fit the observed changes in the dynamic range with the help of a linear fit with 95% confidence intervals, which yields eq 6. Within this equation, DR_non-specific_ is the dynamic range observed for a blank experiment, i.e., by simply adding various concentrations of analyte without the presence of antibody. The Anti_conc_ is the concentration of the antibody, and *M* is a constant or sensitivity of the dynamic range, which is dependent on several factors such as material property and sensor sensitivity. In our case, DR_non-specific_ = 1.496 and *M* = 0.396.
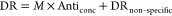
6

Based on our model, eq 6 serves as a fundamental tool in assessing a sensor’s ability to accurately capture a wide range of analyte concentrations, enabling researchers to determine the concentration of the capture antibody for tuning the dynamic range of the sensor.

## Conclusions

In summary, the findings from this research highlight the importance of carefully considering the antibody concentrations in immunoassays to mitigate the prozone effect. By understanding the kinetics of the prozone effect and optimizing the assay conditions, we can enhance the accuracy and sensitivity of LFDs. For example, in our experiments used to develop the model, we have demonstrated that the overall dynamic range improves as the capture antibody concentration increases from 0.1 to 2 mg/mL. Therefore, our developed model and mathematical equations can contribute to the development of more robust and reliable immunoassays, enabling better detection and diagnosis of various diseases in clinical settings. Further research in this area can lead to advancements in sandwich immunoassays used in diagnostic technologies and improve patient care.

## Material and Methods

### Materials

Anti-CRP monoclonal capture (C6) and anti-CRP monoclonal detection (C5) antibodies were purchased from HyTest Ltd. (Finland) and CRP antigen (30C-CP1000U) was purchased from Fitzgerald (USA). The nitrocellulose membrane (CN140) was obtained from Sartorius (Germany), the absorbent pad (A222) was purchased from Ahlstrom (The Netherlands), and the backing card (width 80 mm and thickness 0.015 in.) was purchased from DCN (USA). Heterobifunctional thiol PEG carboxyl (HS-PEG-COOH, 5 kDa) was purchased from JenKem Technology (USA). Chloroauric acid, sodium citrate tribasic dihydrate, *N*-(3-(dimethylamino)propyl)-*N*-ethylcarbodiimide hydrochloride (EDC), *N*-hydroxysuccinimide (NHS), phosphate-buffered saline (PBS), Tris-buffered saline (TBS), sodium phosphate monobasic monohydrate, 2-(*N*-morpholino)ethanesulfonic acid (MES), hydroxylamine, Tween 20, sodium phosphate dibasic monohydrate, and Amicon purification filters were purchased from Sigma-Aldrich (UK).

### AuNP Synthesis and Surface Modification

AuNPs were synthesized using the conventional Turkevich method.^24^ Briefly, 50 mL of chloroauric acid was heated to rapid boiling before being injected with 2 mL of sodium citrate tribasic dihydrate, and a color change from yellow to black to dark red was observed upon the formation of 20 nm diameter AuNPs. To allow for covalent conjugation, the AuNPs surface was modified with 5 mM HS-PEG-COOH. Surface modification was completed by rotating the AuNPs and HS-PEG-COOH mixture for 1 h away from light. Finally, AuNPs–COOH were washed twice via centrifugation to remove excess HS-PEG-COOH and resuspended with deionized water.

### AuNPs–COOH–*anti*-CRP Conjugation

The *anti*-CRP detection antibody was purified before use using an Amicon filter unit to remove amine terminated molecules, which could potentially interfere with the conjugation process. The purified antibody was resuspended in 10 mM potassium phosphate buffer, pH 7.4. In brief, AuNPs–COOH–*anti*-CRP conjugates were prepared by mixing water-soluble EDC and NHS with 50 μL of AuNPs of optical density (OD) 20, 20 μL of detection antibody, and 10 μL of 150 mM MES, pH 5. The resultant solution was incubated for 20 min at room temperature, followed by the addition of 1 μL of hydroxylamine and incubation for a further 10 min. Then, 1 mL of 1 × TBS, 0.05% Tween was added before centrifuging at 10,000 rpm at 20^*o*^C for 10 min. The supernatant was removed, and the pellet of AuNPs–COOH–*anti*-CRP was resuspended in 90 μL of 1 × TBS, 0.05% Tween, and 0.5% casein to obtain a final solution of 10 OD AuNPs–COOH–*anti*-CRP conjugate.

### Fabrication of Half-Dipstick LFD Strips

The *anti*-CRP capture antibody used for the test line was dispensed onto the nitrocellulose (NC) membrane using a BioDot (ZX1010) dispensing platform from BioDot (UK) at a flow rate of 1 μL/cm to obtain a line width of 1 mm. The NC membrane was dried in an oven at 37 °C for 30 min. The dried NC membrane was laminated onto a plastic backing card followed by an absorbent pad with 5 mm overlap on the NC membrane. Finally, strips were cut 5 mm wide and stored in aluminum foil bags with a desiccant until required.

### Analysis of the Signal Output

The CRP antigen was diluted in 10 mM PBS at pH 7.4 buffer to a desired concentration. For half-dipstick LFDs, 10 μL of sample was mixed with 5 μL of AuNPs–COOH–*anti*-CRP OD 5 and 45 μL of running buffer consisting of 10 mM PBS (pH 7.4) and 1% Tween 20. Each capture antibody concentration (0.1, 0.5, 1, 1.5, and 2 mg/mL) was tested in triplicate. After 20 min of incubation, the colorimetric signal at the test line in LFD strip was captured with a Leelu reader (LUMOS-V3-03) from Lumos Diagnostics (USA). The signal intensity of the test line was normalized by subtracting the signal intensity of the bare NC membrane strip with ImageJ software.
